# Restructuring areas, reshaping norms: Denormalizing (e-)cigarette use in Swiss vocational and high schools

**DOI:** 10.18332/tpc/217157

**Published:** 2026-06-18

**Authors:** Anna Morf, Frank Wieber, Dominique Truninger, Gilles Chatelain, Bettina Höchli

**Affiliations:** 1 Department Consumer Behavior and Behavior Change, Institute of Marketing and Management, University of Bern, Bern, Switzerland; 2 School of Health Sciences, Institute of Public Health, Zurich University of Applied Sciences, Winterthur, Switzerland; 3 Department of Psychology, University of Konstanz, Konstanz, Germany; 4 The Behavior Lab Ltd., Oftringen, Switzerland

**Keywords:** adolescents, tobacco and nicotine products, social norms, school environment, denormalization

## Abstract

**INTRODUCTION:**

Adolescents often overestimate peer smoking and vaping, increasing the likelihood of initiation. School environments play a central role in shaping these perceptions. This study therefore aimed to examine whether a school-based environmental intervention modifying the school environment can shift adolescents’ perceived norms and reduce (e-)cigarette use.

**METHODS:**

A quasi-experimental pre-post study was conducted in three Swiss secondary schools (March–June 2024; baseline n=664; follow-up n=884). The intervention comprised a multi-component environmental approach, including the relocation of designated smoking areas to less visible locations, accompanying signage, improvements to non-smoking areas, and informational posters. Perceived peer (e-)cigarette use, and self-reported (e-)cigarette use were reported through online surveys at baseline and at one month post-intervention.

**RESULTS:**

Effects varied across schools. In School 1, perceived prevalence of (e-) cigarette use decreased (cigarettes: β= -0.44; 95% CI: -0.68 – -0.21; e-cigarettes: β= -0.53; 95% CI: -0.80 – -0.25). In School 2, reductions differed by (e-)cigarette use: never and former users reported lower perceived cigarette prevalence at follow-up, whereas occasional and regular users showed no change. Perceived e-cigarette prevalence decreased (β= -0.42; 95% CI: -0.69 – -0.15). In School 3, changes varied by (e-)cigarette use, with regular users reporting lower perceived cigarette prevalence (interaction: β= -1.60; 95% CI: -2.75 – -0.45). Perceived e-cigarette prevalence did not change. Across schools, the intervention did not affect (e-)cigarette use.

**CONCLUSIONS:**

A school-based environmental intervention modifying the school environment may influence adolescents’ descriptive norms, although effects appear to depend on baseline policies and implementation context.

## INTRODUCTION

Adolescence is a critical period for the development of health-related behaviors with long-lasting implications for both the individual and public health^1^. Tobacco use is largely initiated before the age of 18 years^2^. Such an early initiation often leads to long-term addiction and smoking-related health risks^3^. Over the past decades, adolescent cigarette smoking has declined in Europe^4^. However, this positive trend is increasingly challenged by the growing popularity of e-cigarettes, which threatens to reverse progress in tobacco control^5^. This shift in cigarette and e-cigarette use is also evident in Switzerland, where this study was conducted. While recent trends in Switzerland indicate a slight decline in cigarette use among adolescents, the increasing popularity of e-cigarettes remains a concern^6^. According to the most recent Health Behavior in School-aged Children (HBSC) survey, one in four 15-year-olds in Switzerland reported using an e-cigarette in the past month, with e-cigarette use among girls nearly doubling from 2018 to 2022^7^.

This upward trend not only represents a public health concern due to the potential harm of e-cigarette use itself, but also raises concerns about the potential broader renormalization of smoking behaviors. Renormalization refers to a reversal of progress in the denormalization of tobacco use, which is the process of shifting public attitudes and norms to make smoking less socially acceptable^8^. Increased visibility of e-cigarette use may weaken denormalization efforts, potentially making smoking more socially acceptable again^8^. This shift can contribute to higher rates of cigarette smoking, as adolescents who use e-cigarettes are more likely to transition to traditional cigarettes and develop long-term smoking habits^7,9^.

In the context of renormalization of tobacco use among adolescents, social norms play a central role. They strongly influence what is perceived as acceptable and normal within a person’s social environment. An important aspect of social norms concerns how individuals perceive what others typically do, referred to as descriptive norms^10^. These perceptions are an important influence on behavior, as they can be cued by observable actions in the social environment and provide information about what is considered typical or normal^11^.

As cigarette and e-cigarette use become more visible, in public spaces, on social media, and especially in school settings, adolescents may perceive these behaviors as more common and socially acceptable and become more likely to experiment with or adopt these behaviors themselves^12-14^. Given the high addictive potential of e-cigarettes, even occasional or experimental use may result in dependence and long-term nicotine consumption^15^. Greater visibility also leads to misperceptions among adolescents regarding the prevalence and acceptability of smoking and vaping within their peer group^16,17^. Reducing smoking visibility and vaping in school environments may therefore help correct overestimations of smoking prevalence and support healthier social norms among students. As institutions where adolescents spend much of their daily lives, schools are key social contexts where peer behaviors and environmental cues strongly shape normative perceptions^14,18^. Thus, schools represent an ideal setting for tobacco prevention interventions that reinforce non-smoking norms and discourage cigarette and e-cigarette use among students.

Previous research has primarily focused on the implementation and impact of complete smoke-free campus policies or general school smoking bans, and findings suggest that their effectiveness depends on consistent enforcement and contextual adaptation^19-21^. However, less attention has been paid to how broader environmental modifications within schools, including changes to the spatial organization and regulation of smoking and vaping, may influence adolescents’ normative perceptions. This gap is particularly relevant given the rise in adolescent e-cigarette use, as visibility of smoking and vaping may reinforce the social renormalization of tobacco products^8,14^.

To understand how a visibility-reducing intervention can be most effectively implemented, it is important to consider the diversity of school tobacco policies in Switzerland. In Switzerland, school tobacco policies are determined at the cantonal or school level, resulting in considerable variation^22^. Some schools enforce full smoking bans, some have designated smoking areas, and some have no smoking policies. Although strict bans may appear effective, they can be challenging to enforce and may also lead to unintended consequences, such as students smoking off campus in unsupervised areas, showing oppositional behavior, or experiencing increased stigmatisation^23,24^. It is important to note that the present study was conducted before the Swiss Tobacco Products Act (TabPG) came into effect on 1 October 2024, which prohibits the sale of tobacco and electronic cigarettes to individuals under 18 years and restricts advertising in public spaces^25^. As data collection took place in March and June 2024, the findings presented here reflect the situation prior to these regulatory changes.

We conceptualize perceived prevalence, also referred to as the descriptive norm of cigarette and e-cigarette use as a key element of the denormalization process, which refers to reducing the social acceptance and visibility of smoking behaviors^26^. From this perspective, a reduction in perceived prevalence is an important initial step toward denormalization because it signals a shift in adolescents’ perceptions of smoking. Smoking is no longer regarded as common and socially accepted, but rather as less prevalent and, ultimately, less acceptable^14^. Against this background, the present study examined whether a multi-component school-based environmental intervention is associated with changes in adolescents’ perceived prevalence as a key element of the denormalization process.

## METHODS

### Study design

This study employed a quasi-experimental, within-school pre-post design with partially repeated measures, conducted in three Swiss secondary schools as part of the ‘Here We Are’ research project^27^. The study was carried out between March and June 2024. The intervention aimed to reduce the visibility of cigarette and e-cigarette use in school environments and to influence adolescents’ perceptions of social norms surrounding cigarette and e-cigarette use through environmental modifications of the school grounds. To assess these changes, data were collected through baseline (T0) and follow-up (T1) questionnaires. Ethical approval was obtained before data collection, and the study was preregistered.

### Participants

The participants were students aged 14–20 years attending the three participating schools. Recruitment was coordinated through school administration, with the study link distributed via email. Participation was voluntary and responses were anonymous. Participants who did not complete the entire questionnaire or were outside the age limits, were excluded.

### Intervention

The intervention focused on environmental modifications to reduce smoking visibility on school grounds and support denormalization of smoking behaviors. Measures were developed in collaborative workshops with students to ensure that they were perceived as constructive rather than punitive^28^. The school-based environmental intervention, comprised multiple components, including the relocation of designated smoking areas to less visible locations, clarification of boundaries through signage and ground markings, relocation or removal of contradictory elements (e.g. ashtrays), improvements to non-smoking areas (e.g. seating, sunshades, outdoor games), and the installation of smoking and vaping cessation posters and stress-management materials.

Implementation differed across schools depending on the existing policy context and spatial conditions. In School 1, designated smoking areas were newly established in peripheral locations, whereas in School 2 existing smoking areas were retained but clarified, and in School 3, new designated smoking areas were established. However, peripheral placement was not possible due to spatial constraints. School-specific environmental modifications are summarized in Table 1.

**Table 1:** Overview of school-specific intervention components

*Component*	*School 1*	*School 2*	*School 3*
**Policy context**	No prior tobacco policy	Designated smoking areas in place	Smoking prohibited only in central courtyard
**Designated smoking areas**	Newly established in peripheral locations; ground markings and signage implemented; ashtrays relocated; smoking and vaping cessation poster displayed	Existing areas retained; boundaries clarified; contradictory elements (e.g. ashtrays in non-smoking areas) removed or covered; smoking and vaping cessation poster displayed	Newly designated areas; peripheral placement not possible due to spatial constraints; markings and signage implemented; umbrellas provided; smoking and vaping cessation poster displayed
**Non-smoking areas**	*Outdoor*: seating, sunshades, outdoor games *Indoor*: board games, anti-stress balls, stress-management poster	*Outdoor*: seating and sunshades *Indoor*: board games, anti-stress balls, stress-management poster	*Outdoor*: markings only *Indoor*: board games, anti-stress balls, stress-management poster

The table summarizes the multi-component environmental intervention implemented at each participating school. All schools received a standardized smoking and vaping cessation poster; other components were adapted to the local policy context and spatial constraints.

Implementation was monitored through school visits to ensure compliance, and impressions of acceptance and feedback were collected through informal conversations with students and staff.

### Measures

Perceived descriptive norms for cigarette and e-cigarette use were measured using the question: ‘Out of every 10 people your age, how many do you think smoke (cigarettes/e-cigarettes) regularly?’. Responses ranged from 0 to 10 and were analyzed as continuous variables. This item was adapted from Agaku et al.^29^. Current smoking status was measured by asking participants ‘Do you smoke?’, with four response options: never smoked, smoke occasionally, smoke regularly, and no longer smoke. Participants who reported current smoking were asked a follow-up question regarding the product type (cigarettes, e-cigarettes, or both). Students reported age, gender, school year, and expected graduation year. Gender was reported as male, female, or diverse. All variables were assessed at baseline and at follow-up.

### Procedure

The baseline survey (T0) was distributed via email to all students by the schools in early March 2024. The intervention was implemented in early May 2024. Prior to implementation, students received another email from the schools informing them about the new smoking policy and changes on the school grounds. The follow-up survey (T1) was conducted approximately one month after intervention rollout in early June 2024 and was again sent to all students at the three participating schools. Participation was voluntary, and the surveys were completed online and anonymously. Because a subset of participants completed both the pre- and post-intervention surveys, the dataset includes repeated measures for some individuals. To identify responses from the same individual across time points while maintaining anonymity, participants created an anonymous identification code by answering a few simple, non-identifying questions.

After completing the survey, participants had the option to enter a prize draw (T0: voucher for Galaxus, a Swiss online retailer; T1: iPad, cinema vouchers). Contact information for the prize draw was collected on a separate website to maintain anonymity. Notably, more students participated at follow-up than at baseline, possibly reflecting greater interest in the topic after policy implementation or differences in the prize draw incentives.

### Statistical analysis

Descriptive statistics were first computed to summarize sample characteristics and perceived cigarette and e-cigarette prevalence at baseline and follow-up. To examine the impact of the intervention on adolescents’ perceptions of smoking prevalence, linear mixed-effects models were computed separately for each school and for each product type (cigarettes, e-cigarettes). All models included fixed effects for time point (baseline vs follow-up) and smoking status (never, former, occasional, regular), and a random intercept for participant ID to account for repeated measures and individual differences in baseline perceptions. Never smokers served as the reference category.

Mixed-effects models were fitted using all available observations, as participation was open at both time points.

For each outcome, a reduced model with only main effects was compared to a full model including the interaction between time point and smoking status using likelihood ratio chi-squared tests. When the interaction did not improve model fit, the reduced model was retained and only the overall intervention effect reported. When the interaction significantly improved fit, the full model was retained and estimated marginal means (EMMs) with pairwise contrasts and standardized effect sizes (Cohen’s d) were calculated.

As a sensitivity analysis, all mixed-effects models were additionally adjusted for age and gender; the results of these analyses are reported in the Supplementary file. Chi-squared tests were also conducted to examine whether the intervention affected smoking status distributions across time. All analyses were performed using R, and statistical significance was set at p<0.05.

## RESULTS

At baseline (T0), 664 students participated across the three schools, and 884 students completed the follow-up survey (T1). Sample characteristics by school and time point are summarized in Table 2. Mean age ranged from 16.3 to 18.0 years across schools and time points. Across all three schools, intervention effects on perceived cigarette and e-cigarette prevalence were examined using linear mixed-effects models. For each outcome, full models including interaction terms were compared with reduced models without interactions to determine the best-fitting specification. Model comparisons and detailed outputs are provided in the Supplementary file.

**Table 2:** Sample characteristics by school and time point

*School*	*Time point*	*Observations*	*Age (years)*		*Males*	*Females*	*Diverse*
			*Mean*	*(SD)*			
**School 1**	T0	266	16.305	1.143	75	185	6
	T1	420	17.005	1.382	139	278	3
**School 2**	T0	297	17.976	1.334	90	204	3
	T1	379	17.879	1.220	112	264	3
**School 3**	T0	101	17.683	1.288	50	50	1
	T1	85	17.671	1.276	38	47	0

T0: baseline assessment. T1: follow-up assessment. Gender categories reflect self-reported gender.

### School 1

**Figure 1:**
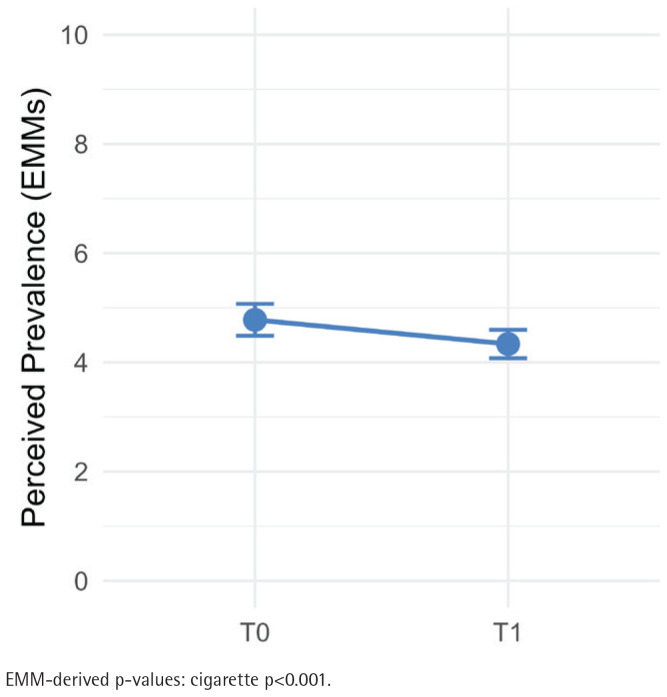
Perceived prevalence of cigarette use among students from School 1 at baseline and follow-up. Estimated marginal means (EMMs) of perceived prevalence of cigarette use (0–10 peers) at baseline (T0) and follow-up (T1), with 95% confidence intervals

In School 1, model comparisons showed that, for both perceived cigarette and e-cigarette prevalence, reduced models without interaction terms provided the best fit. For perceived cigarette prevalence, the model revealed a significant overall intervention effect (β= -0.44; 95% CI: -0.68 – -0.21; p<0.001) (Figure 1). Occasional smokers (β=0.79; 95% CI: 0.37–1.22; p<0.001) and regular smokers (β=0.96; 95% CI: 0.34–1.57: p=0.002) reported higher perceived prevalence at baseline than never smokers, whereas former smokers did not differ significantly (β=0.37; 95% CI: -0.27–1.01; p=0.25).

For perceived e-cigarette prevalence, the reduced model also showed a significant main effect of the intervention (β= -0.53; 95% CI: -0.80 – -0.25; p<0.001) (Supplementary file Figure S1), with no reliable baseline differences between smoking groups.

Overall, the intervention was associated with a reduction in perceived cigarette and e-cigarette prevalence across the student population. A chi-squared test confirmed no significant effect of the intervention on smoking status [χ^2^(3)=2.39; n=686; p=0.50].

### School 2

**Figure 2:**
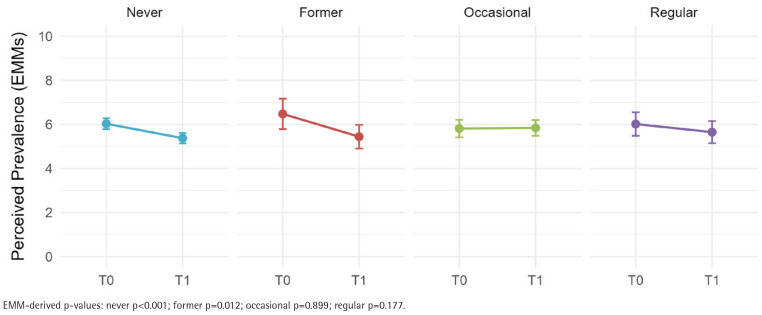
Perceived prevalence of cigarette use among students from School 2 by smoking status at baseline and follow-up. Estimated marginal means (EMMs) of perceived prevalence of cigarette use (0–10 peers) at baseline (T0) and follow-up (T1), with 95% confidence intervals

In School 2, the full model including the interaction provided the best fit for perceived cigarette prevalence. The model revealed a significant overall intervention effect for never smokers, who reported lower perceived cigarette prevalence at follow-up compared with baseline (β= -0.66; 95 % CI: -0.92 – -0.40; p<0.001). The interaction between time point and smoking status was significant (β=0.69; 95 % CI: 0.17–1.20; p=0.009), indicating that changes varied across smoking groups. Estimated marginal means showed significant reductions for never smokers (Δ=0.66; p<0.001; d=0.73) and former smokers (Δ=1.02; p=0.011; d=1.11), whereas occasional (Δ= -0.03; p=0.90; d= -0.03) and regular smokers (Δ=0.37; p=0.18; d=0.41) showed no significant change (Figure 2).

For perceived e-cigarette prevalence, model comparison indicated that the interaction terms were not significant and did not improve fit. The reduced model was therefore retained. This model showed a significant main effect of time point, with lower perceived e-cigarette prevalence at follow-up (β= -0.42; 95% CI: -0.69 – -0.15; p=0.002) (Supplementary file Figure S2). Smoking status was not significantly associated with perceived e-cigarette prevalence.

Overall, the intervention was associated with a reduction in perceived cigarette and e-cigarette prevalence among students in School 2. A chi-squared test confirmed no significant effect of the intervention on smoking status [χ^2^(3)=1.57; n=676; p=0.67].

### School 3

**Figure 3:**
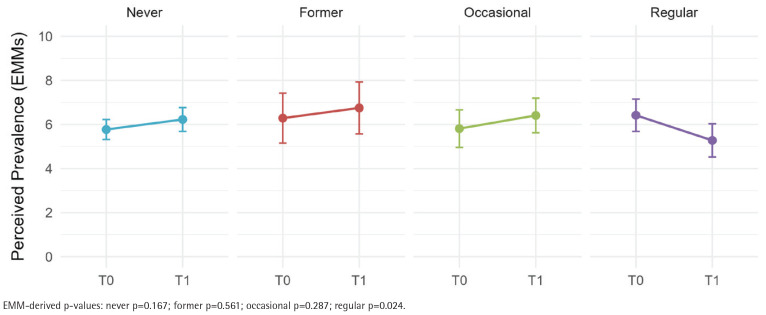
Perceived prevalence of cigarette use among students from School 3 by smoking status at baseline and follow-up. Estimated marginal means (EMMs) of perceived prevalence of cigarette use (0–10 peers) at baseline (T0) and follow-up (T1), with 95% confidence intervals

For school 3, the analyses of the linear mixed-effects models indicated that the full model including the interaction between time point and smoking status provided a significantly better fit than the reduced model. Therefore, the full model was retained for the main analyses. For perceived cigarette prevalence, the main effect of time point indicated that, among never smokers, perceived cigarette prevalence did not change significantly from baseline to follow-up (β=0.46; 95% CI: -0.18–1.09; p=0.16) (Figure 3). However, a significant interaction emerged: regular smokers reported a larger decrease in perceived cigarette prevalence compared with never smokers (β= -1.60; 95% CI: -2.75 – -0.45; p=0.007). Estimated marginal means showed that perceived cigarette prevalence decreased among regular smokers (Δ= -1.14; p=0.024; d= -0.98), whereas values for never (Δ=0.46; p=0.17; d=0.39), former (Δ=0.46; p=0.56; d=0.40), and occasional smokers (Δ=0.60; p=0.29; d=0.51) showed no significant change.

For perceived e-cigarette prevalence, model comparison indicated that the reduced model provided the better fit. This model showed no significant intervention effect (β= 0.29; 95% CI: -0.23–0.82; p=0.27) (Supplementary file Figure S3) and no significant baseline differences between smoking groups.

Overall, the analyses for School 3 indicated an intervention effect limited to regular smokers. A chi-squared test confirmed no significant effect of the intervention on smoking status [χ^2^(3)=2.64; n=190; p=0.45].

## DISCUSSION

This exploratory study examined whether a multi-component environmental intervention modifying school environments was associated with changes in adolescents’ perceptions of smoking-related norms. Perceived prevalence of cigarette and e-cigarette use decreased in School 1, and for e-cigarettes also in School 2. For cigarettes, reductions in School 2 were limited to never and former users and in School 3 to regular users. Across schools, no short-term changes in self-reported (e-)cigarette use were observed. Taken together, these findings suggest that environmental interventions implemented in school settings may influence adolescents’ descriptive norms, with effects varying by baseline policy context and students’ (e-)cigarette use.

Our findings are consistent with prior research showing that adolescents’ perceptions of peer smoking are influenced by environmental context but extend this evidence to a real-world school intervention context, where physical environments were actively modified^14,17^. The observed pattern also aligns with previous research showing that adolescents tend to overestimate peer smoking and vaping prevalence^30-32^. From a social norms perspective, descriptive norms reflect beliefs about what is typical among peers and are shaped by everyday observations and contextual cues in adolescents’ social environments^10,11^. Changes in perceived prevalence may therefore represent an early indicator of normative shifts that can precede behavioral change, particularly in the context of short follow-up periods.

Differences across schools highlight the importance of baseline policy context for understanding why environmental interventions may have differential effects, a pattern also described in prior research on school tobacco control and policy implementation^19-21,23^. In School 1, where no smoking policies or designated smoking areas existed prior to the intervention, the introduction of clearly defined smoking and non-smoking areas, combined with broader environmental modifications, represented a substantial change to the school environment. These changes were associated with reduced visibility of smoking on school grounds and may have signaled lower social acceptance of cigarette and e-cigarette use, which may help explain the consistent reductions in perceived cigarette and e-cigarette prevalence across student groups. This interpretation is consistent with prior research indicating that both school-level tobacco control and peer contexts influence adolescents’ perceptions of smoking norms^33,34^.

In School 2, designated smoking areas were already in place before the intervention. Here, changes primarily involved clarifying where smoking was permitted, removing contradictory elements such as ashtrays in non-smoking areas, and complementing these adjustments with further environmental modifications. In line with research suggesting that the impact of school tobacco measures depends on their clarity and coherence, these combined changes were associated with a reduction in perceived e-cigarette prevalence, whereas effects on perceived cigarette prevalence were confined to never and former users^19-21^. This pattern is compatible with previous findings indicating that adolescents who do not smoke may be more responsive to contextual signals when estimating peer behavior, whereas regular users rely more strongly on their own behavior and peer networks33,34.

In School 3, spatial constraints limited the relocation of designated smoking areas away from central locations. Despite broader environmental changes, perceived prevalence decreased only among regular cigarette users. This suggests that students directly affected by newly enforced restrictions were responsive to changes in their immediate smoking environment, even though visibility of smoking and vaping on campus remained largely unchanged. This pattern mirrors findings from prior work suggesting that the effects of school tobacco measures may be strongest among students who are directly affected by rule changes, even when overall school-wide exposure remains similar^23^. Taken together, these school-specific findings indicate that environmental interventions interact with existing policies and spatial constraints, shaping both the strength and reach of their effects on adolescents’ normative perceptions.

Informal observations during school visits further suggested differences in acceptability and perceived feasibility across schools. In School 1, the newly established measures appeared to face little resistance, whereas in School 3 students expressed concerns about the size and lack of shelter of designated smoking areas, and in School 2 some students reported dissatisfaction with their distance from the main entrance. While not systematically assessed, these contextual factors may be relevant for interpreting variation in implementation and observed effects across schools.

The findings indicate that changes to school environments were associated with adolescents’ normative perceptions even in the absence of comprehensive smoke-free school policies. Prior research has largely focused on comprehensive smoke-free campus policies or general smoking bans, with evidence suggesting that their effectiveness depends on enforcement, contextual adaptation, and acceptability^19-21^. While such policies can reduce exposure, they may also be difficult to implement or lead to unintended consequences, such as smoking displacement or oppositional behavior^23^. The present study adds to this literature by suggesting that a multi-component environmental approach, including the relocation of designated smoking areas to less visible locations, accompanying signage, improvements to non-smoking areas, and informational posters, may also influence descriptive norms, particularly when it introduces clear and coherent signals in settings with limited prior regulation.

At the same time, the modest and context-dependent effects observed here underscore that environmental interventions are not universally effective and should not be expected to produce immediate behavioral change. Their potential value may lie in contributing to gradual norm shifts that, over time and in combination with other measures, support denormalization processes, as conceptualized in prior work on tobacco control and renormalization^8,14^.

### Limitations

This study has several limitations that should be considered when interpreting the findings. First, the quasi-experimental pre-post design without a control group limits the ability to draw causal conclusions, as observed changes cannot be unequivocally attributed to the intervention. In addition, the short follow-up period of approximately one-month limits conclusions regarding the persistence of changes in perceived descriptive norms and their potential translation into smoking or vaping behavior.

Second, while overall sample sizes were adequate, subgroup sizes were modest, which reduced statistical power. Third, all outcomes relied on self-reported data and may be affected by recall bias. However, as the primary outcomes focused on perceived prevalence, subjective assessments remain theoretically relevant given the established role of perceptions in shaping behavior^10^. Nevertheless, self-reports may also introduce misclassification bias, particularly with respect to smoking and vaping behavior.

Participation was voluntary and surveys were distributed via email, which may have introduced self-selection bias. Nevertheless, the observed variability in perceived norms across schools and subgroups suggests that a ceiling effect is unlikely. Residual confounding cannot be ruled out, as unmeasured individual- or school-level factors may have influenced the results.

Furthermore, the intervention comprised multiple environmental components implemented simultaneously, precluding disentanglement of the specific contribution of individual elements. The findings should therefore be interpreted as reflecting the combined effect of a multi-component environmental approach rather than the impact of any single measure. Although observed effect sizes were relatively small, they may still be meaningful at the population level, as modest shifts in perceived norms can accumulate over time.

Finally, generalizability may be limited, as the study was conducted in three Swiss secondary schools within a specific regulatory and cultural context prior to the implementation of the revised Swiss Tobacco Products Act. Future research should employ controlled designs, longer follow-up periods, and larger samples to assess the durability of normative changes and their association with behavioral outcomes.

## CONCLUSIONS

This exploratory study examined whether a multi-component school-based environmental intervention modifying school environments is associated with changes in adolescents’ perceived smoking-related norms. Across the three participating schools, perceived prevalence of cigarette and e-cigarette use declined in some contexts, although the direction and magnitude of effects varied by baseline policy context and students’ (e-)cigarette use. Overall, the findings suggest that environmental modifications to school grounds, including changes to how and where smoking and vaping are regulated and encountered, may contribute to shifts in adolescents’ normative perceptions. These effects appear to be highly context dependent, with stronger and more consistent effects observed in settings where regulations were newly introduced or substantially clarified.

Importantly, even modest changes in perceived norms may be meaningful, as they can signal evolving social expectations and potentially reduce the likelihood of smoking or vaping initiation among adolescents. Schools and, more broadly, other public spaces with high adolescent presence and exposure, such as playgrounds and sports facilities, represent promising settings for environmental approaches that aim to reinforce non-smoking norms through coherent and context-sensitive regulation of smoking-related spaces.

## Supplementary Material



## Data Availability

The data supporting this research are available from the following source: https://osf.io/aebdj/?view_only=ceee00436dd44fd5a69b8d97ee5810df.
