# Supplementary material for: Restructuring areas, reshaping norms: Denormalizing (e-)cigarette use in Swiss vocational and high schools
Source: Tob Prev Cessat. 2026 Jun 18;12:10.18332/tpc/217157. doi: 10.18332/tpc/217157 (PMC13277420; doi:10.18332/tpc/217157)
Supplement: Supplementary file 1 [file TPC-12-29-s1.pdf]

Supplementary File

This Supplementary File provides additional figures, descriptive statistics, and full model outputs for each participating school (School 1, School 2, and School 3). For each school, the Supplement contains, model outputs, model comparisons, and where applicable, estimated pairwise contrasts. All analyses were conducted in R (version 4.3.0) using the packages lme4, lmerTest, emmeans, and sjPlot. Linear mixed effects models were fitted with random intercepts for participant identification.

Table of Contents

Supplementary File 1

1 Figures for Perceived Prevalence of E-Cigarettes 2

1.1 School 1 2

1.2 School 2 3

1.3 School 3 3

2 School 1 4

2.1 Perceived Cigarette Prevalence School 1 4

2.1.1 Model Output 4

2.1.2 Model comparison 4

2.1.3 Sensitivity analysis 5

2.2 Perceived E-Cigarette Prevalence School 1 5

2.2.1 Model Output 5

2.2.2 Model Comparison 6

2.2.3 Sensitivity analysis 6

3 School 2 7

3.1 Perceived Cigarette Prevalence School 2 7

3.1.1 Model Output 7

3.1.2 Model Comparison 7

3.1.3 Estimated Pairwise Contrasts 8

3.1.4 Sensitivity analysis 8

3.2 Perceived E-Cigarette Prevalence School 2 8

3.2.1 Model Output 8

3.2.2 Model Comparison 9

3.2.3 Sensitivity analysis 9

4 School 3 10

4.1 Perceived Cigarette Prevalence School 3 10

4.1.1 Model Output 10

4.1.2 Model Comparison 11

4.1.3 Estimated Pairwise Contrasts 11

4.1.4 Sensitivity analysis 11
Perceived E-Cigarette Prevalence School 3 12
4.1.5 Model Comparison 12
4.1.6 Model Output 12
4.1.7 Sensitivity analysis 13

1 Figures for Perceived Prevalence of E-Cigarettes

1.1 School 1

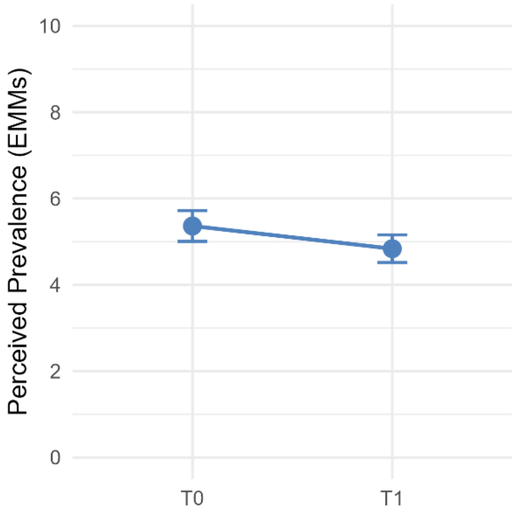

Figure S1. Perceived prevalence of e-cigarette use among students from School 1 at baseline and follow-up (N = 686). Estimated marginal means (EMMs) of perceived prevalence of e-cigarette use (0–10 peers) at baseline (T0) and follow-up (T1), with 95% confidence intervals. EMM-derived *p*-values: *p* = <.001.

1.2 School 2

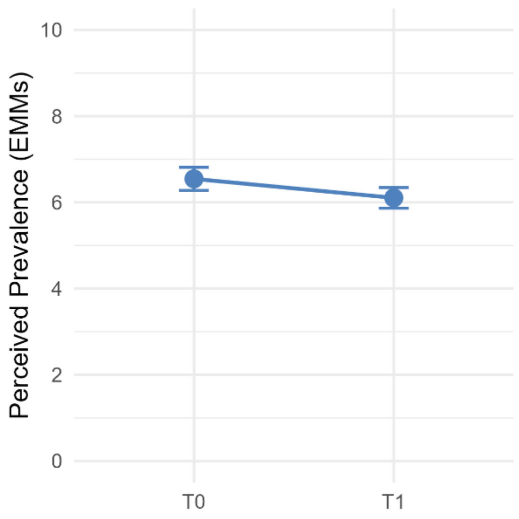

Figure S2. Perceived prevalence of e-cigarette use among students from School 2 at baseline and follow-up. Estimated marginal means (EMMs) of perceived prevalence of e-cigarette use (0–10 peers) at baseline (T0) and follow-up (T1) (N = 676), with 95% confidence intervals. EMM-derived p-value: p =.003.

1.3 School 3

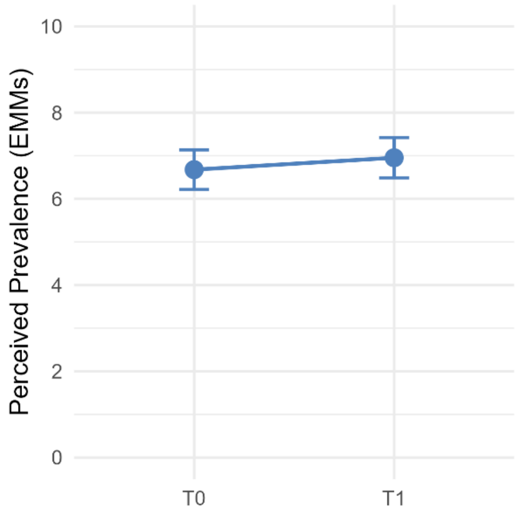

Figure S3. Perceived prevalence of e-cigarette use among students from School 3 at baseline and follow-up. Estimated marginal means (EMMs) of perceived prevalence of e-cigarette use (0–10 peers) at baseline (T0) and follow-up (T1) **(**N = 186)**,** with 95% confidence intervals. EMM-derived *P* = .283.

2 School 1

2.1 Perceived Cigarette Prevalence School 1

2.1.1 Model Output

Table S1. Reduced linear mixed-effects model predicting perceived cigarette prevalence in School 1 at baseline and follow-up (N = 686).

	Perceived Prevalence (Cigarette)			
Predictors	*Estimates*	*CI*	*Statistic*	*p*
(Intercept)	4.25	4.02 – 4.47	37.13	**<0.001**
Timepoint [T1]	-0.44	-0.68 – -0.21	-3.68	**<0.001**
(E-)cigarette Use [Former]	0.37	-0.27 – 1.01	1.15	0.253
(E-)cigarette Use [Occasional]	0.79	0.37 – 1.22	3.69	**<0.001**
(E-)cigarette Use [Regular]	0.96	0.34 – 1.57	3.05	**0.002**
**Random Effects**				
σ^2	1.43			
τ_{00} Identification	2.10			
ICC	0.59			
N Identification	563			
Observations	686			
Marginal R^2 / Conditional R^2	0.041 / 0.611			

Note. CI = confidence interval; ICC = intraclass correlation coefficient. Never-smokers served as the reference category.

2.1.2 Model comparison

Table S2. Likelihood-ratio test comparing full and reduced model predicting perceived cigarette prevalence in School 1.

**Model**	**df**	**AIC**	**BIC**	**logLik**
Reduced Model	7	2766.5	2798.2	-1376.3
Full Model	10	2771.9	2817.2	-1376.0

Note. The full model including the interaction between *Timepoint* and *(E-)cigarette_Use* did not significantly improve model fit compared with the reduced model ($\chi^2(3) = 0.62$, $p = .89$). The reduced model was therefore retained. AIC = Akaike information criterion; BIC = Bayesian information criterion.

2.1.3 Sensitivity analysis

Table S3. Sensitivity analysis for School 1 for perceived cigarette prevalence (adjusted for age and gender)

Section	Comparison / Predictor	Estimate / Test	95% CI	p
**Model comparison (ML)**	Timepoint × (E-)cigarette Use	$\chi^2(3) = 0.67$	–	0.88
**Final adjusted model (REML)**	Timepoint (post vs pre)	−0.57	−0.80 to −0.33	<0.001

Note. Linear mixed-effects models with a random intercept for participant. Age was centered. Model comparison was conducted using maximum likelihood; final estimates were obtained using restricted maximum likelihood. CI = confidence interval. Never-smokers served as the reference category.

2.2 Perceived E-Cigarette Prevalence School 1

2.2.1 Model Output

Table S4. Reduced linear mixed-effects model predicting perceived e-cigarette prevalence in School 1 at baseline and follow-up (N = 686).

	**Perceived Prevalence (E-Cigarette)**			
Predictors	*Estimates*	*CI*	*Statistic*	*p*
(Intercept)	5.12	4.85 – 5.40	36.85	**<0.001**
Timepoint [T1]	-0.53	-0.80 – -0.25	-3.75	**<0.001**
(E-)cigarette Use [Former]	0.30	-0.47 – 1.08	0.78	0.438
(E-)cigarette Use [Occasional]	0.33	-0.19 – 0.85	1.25	0.212
(E-)cigarette Use [Regular]	0.32	-0.44 – 1.09	0.83	0.404
**Random Effects**				
σ^2	1.80			
τ_{00} Identification	3.66			
ICC	0.67			
N Identification	563			
Observations	686			
Marginal R^2 / Conditional R^2	0.015 / 0.676			

Note. CI = confidence interval; ICC = intraclass correlation coefficient; σ^2 = residual variance; τ_{00} = variance of the random intercept. Never-smokers served as the reference category.

2.2.2 Model Comparison

Table S5. Likelihood-ratio test comparing full and reduced models predicting perceived e-cigarette prevalence in School 1 at baseline and follow-up (N = 686).

Model	df	AIC	BIC	logLik
Reduced Model	7	3046.0	3077.7	-1516.0
Full Model	10	3047.6	3092.9	-1513.8

Note. The full model including the interaction between *Timepoint* and *(E-)cigarette_Use* did not significantly improve model fit compared with the reduced model ($\chi^2(3) = 4.42$, $p = .22$). AIC = Akaike information criterion; BIC = Bayesian information criterion.

2.2.3 Sensitivity analysis

Table S6. Sensitivity analysis for School 1 perceived e-cigarette prevalence (adjusted for age and gender)

Section	Comparison / Predictor	Estimate / Test	95% CI	p
**Model comparison (ML)**	Timepoint × (E-)cigarette Use	$\chi^2(3) = 3.84$	–	0.28
**Final adjusted model (REML)**	Timepoint (post vs pre)	−0.49	−0.76 to −0.21	<0.001

Note. Linear mixed-effects models with a random intercept for participant identification; adjusted for age (centered) and gender. Model comparison was conducted using maximum likelihood; final estimates were obtained using restricted maximum likelihood. CI = confidence interval. Never-smokers served as the reference category.

3 School 2

3.1 Perceived Cigarette Prevalence School 2

3.1.1 Model Output

Table S7. Full linear mixed-effects model predicting perceived cigarette prevalence in School 2 at baseline and follow-up (N = 676).

	Perceived Prevalence (Cigarette)			
Predictors	*Estimates*	*CI*	*Statistic*	*p*
(Intercept)	6.03	5.78 – 6.28	47.78	**<0.001**
Timepoint [T1]	-0.66	-0.92 – -0.40	-4.93	**<0.001**
(E-)cigarette Use [Former]	0.45	-0.28 – 1.17	1.21	0.227
(E-)cigarette Use [Occasional]	-0.22	-0.67 – 0.24	-0.94	0.346
(E-)cigarette Use [Regular]	-0.01	-0.60 – 0.57	-0.05	0.963
Timepoint [T1] x (E-)cigarette Use [Former]	-0.38	-1.23 – 0.47	-0.87	0.385
Timepoint [T1] x (E-)cigarette Use [Occasional]	0.69	0.17 – 1.20	2.61	**0.009**
Timepoint [T1] x (E-)cigarette Use [Regular]	0.29	-0.30 – 0.88	0.96	0.339
**Random Effects**				
σ^2	0.80			
τ_{00} Identification	2.95			
ICC	0.79			
N Identification	558			
Observations	676			
Marginal R^2 / Conditional R^2	0.024 / 0.791			

Note. CI = confidence interval; ICC = intraclass correlation coefficient; σ^2 = residual variance; τ_{00} = variance of the random intercept. Never-smokers served as the reference category.

3.1.2 Model Comparison

Table S8. Likelihood-ratio test comparing full and reduced models predicting perceived cigarette prevalence in School 2 at baseline and follow-up (N = 676).

Model	df	AIC	BIC	logLik
Reduced Model	7	2713.7	2745.3	-1349.8
Full Model	10	2711.2	2756.3	-1345.6

Note. The full model including the interaction between *Timepoint* and *(E-)cigarette_Use* significantly improved model fit compared with the reduced model ($\chi^2(3) = 8.5$, $p = .04$). AIC = Akaike information criterion; BIC = Bayesian information criterion.

3.1.3 Estimated Pairwise Contrasts

Table S9. Estimated pairwise contrasts (baseline − follow-up) for perceived cigarette prevalence in School 2 (N = 676).

(E-)cigarette Use	Contrast	Estimate	95% CI	SE	df	t-ratio	p-value
Never	T0 – T1	0.66	0.39 –0.92	0.134	219	4.91	**< 0.001**
Former	T0 – T1	1.03	0.23 –1.84	0.410	360	2.52	**0.012**
Occasional	T0 – T1	-0.03	−0.47– 0.42	0.225	241	-0.13	0.898
Regular	T0 – T1	0.37	−0.17 – 0.91	0.272	195	1.35	0.177

Note. Estimates represent differences between baseline (T0) and follow-up (T1). Positive values indicate lower perceived prevalence at follow-up. Confidence intervals are 95% and based on the Kenward–Roger degrees-of-freedom method. Never-smokers served as the reference category.

3.1.4 Sensitivity analysis

Table S10. Sensitivity analysis for School 2 perceived cigarette prevalence (adjusted for age and gender)

Section	Comparison / Predictor	Estimate / Test	95% CI	p
**Model comparison (ML)**	Timepoint × (E-)cigarette Use	$\chi^2(3) = 9.67$	–	0.022
**Final adjusted model (REML)**	Timepoint (post vs pre)*	−0.49	−0.69 to −0.29	<0.001

Note. Linear mixed-effects models with a random intercept for participant identification; adjusted for age (centered) and gender. Model comparison was conducted using maximum likelihood. The timepoint × (e-)cigarette use interaction was statistically significant, indicating that changes in perceived cigarette prevalence over time differed by (e-)cigarette use. Final estimates were obtained using restricted maximum likelihood. Never-smokers served as the reference category.

3.2 Perceived E-Cigarette Prevalence School 2

3.2.1 Model Output

Table S11. Reduced linear mixed-effects model predicting perceived e-cigarette prevalence in School 2 at baseline and follow-up (N = 676).

	Perceived Prevalence (E-Cigarette)			
Predictors	*Estimates*	*CI*	*Statistic*	*p*
(Intercept)	6.49	6.21 – 6.76	46.80	**<0.001**

Timepoint [T1]	-0.42	-0.69 – -0.15	-3.03	**0.002**
(E-)cigarette Use [Former]	0.08	-0.50 – 0.67	0.28	0.778
(E-)cigarette Use [Occasional]	0.29	-0.13 – 0.71	1.36	0.173
(E-)cigarette Use [Regular]	-0.07	-0.63 – 0.48	-0.26	0.794
**Random Effects**				
σ^2	1.89			
τ_{00} Identification	2.93			
ICC	0.61			
N Identification	558			
Observations	676			
Marginal R^2 / Conditional R^2	0.012 / 0.612			

Note. CI = confidence interval; ICC = intraclass correlation coefficient; σ^2 = residual variance; τ_{00} = variance of the random intercept. Never-smokers served as the reference category.

3.2.2 Model Comparison

Table S12. Likelihood-ratio test comparing full and reduced models predicting perceived e-cigarette prevalence in School 2 at baseline and follow-up (N = 676).

Model	df	AIC	BIC	logLik
Reduced Model	7	2936.9	2968.5	-1461.5
Full Model	10	2941.4	2986.5	-1460.7

Note. The full model including the interaction between *Timepoint* and *(E-)cigarette_Use* significantly improvde model fit compared with the reduced model ($\chi^2(3)$ = 1.54, p = .77). AIC = Akaike information criterion; BIC = Bayesian information criterion.

3.2.3 Sensitivity analysis

Table S13. Sensitivity analysis for School 2 perceived e-cigarette prevalence (adjusted for age and gender)

Section	Comparison / Predictor	Estimate / Test	95% CI	p
**Model comparison (ML)**	Timepoint × (E-)cigarette Use	$\chi^2(3)$ = 2.08	–	0.56
**Final adjusted model (REML)**	Timepoint (post vs pre)	−0.42	−0.68 to −0.15	0.002

Note. Linear mixed-effects models with a random intercept for participant identification; adjusted for age (centered) and gender. Model comparison was conducted using maximum likelihood. The timepoint × (e-)cigarette use interaction was not supported, indicating that changes over time did not differ by (e-)cigarette use. The main effect of timepoint remained statistically significant after adjustment, demonstrating robustness of the results. Never-smokers served as the reference category.

4 School 3

4.1 Perceived Cigarette Prevalence School 3

4.1.1 Model Output

Table S14. Full linear mixed-effects model predicting perceived cigarette prevalence in School 3 at baseline and follow-up (N = 186).

	**Perceived Prevalence (Cigarette)**			
Predictors	*Estimates*	*CI*	*Statistic*	*p*
(Intercept)	5.77	5.32 – 6.22	25.29	**<0.001**
Timepoint [T1]	0.46	-0.18 – 1.09	1.43	0.156
(E-)cigarette Use [Former]	0.52	-0.70 – 1.73	0.84	0.403
(E-)cigarette Use [Occasional]	0.04	-0.91 – 0.99	0.08	0.933
(E-)cigarette Use [Regular]	0.65	-0.21 – 1.50	1.50	0.136
Timepoint [T1] × (E-)cigarette Use [Former]	0.01	-1.66 – 1.68	0.01	0.991
Timepoint [T1] × (E-)cigarette Use [Occasional]	0.14	-1.11 – 1.39	0.22	0.823
Timepoint [T1] × (E-)cigarette Use [Regular]	-1.60	-2.75 – -0.45	-2.74	**0.007**
**Random Effects**				
σ^2	1.36			
τ_{00} Identification	1.69			
ICC	0.55			
N Identification	161			
Observations	186			
Marginal R^2 / Conditional R^2	0.047 / 0.575			

Note. CI = confidence interval; ICC = intraclass correlation coefficient; σ^2 = residual variance; τ_{00} = variance of the random intercept. Never-smokers served as the reference category.

4.1.2 Model Comparison

Table S15. Likelihood-ratio test comparing full and reduced models predicting perceived cigarette prevalence in School 3 at baseline and follow-up (N = 186).

Model	df	AIC	BIC	logLik
Reduced Model	7	740.24	762.82	-363.12
Full Model	10	737.46	769.71	-358.73

Note. The full model including the interaction between *Timepoint* and *(E-)cigarette_Use* significantly improved model fit compared with the reduced model ($\chi^2(3) = 8.8$, $p = .03$). AIC = Akaike information criterion; BIC = Bayesian information criterion.

4.1.3 Estimated Pairwise Contrasts

Table S16. Estimated pairwise contrasts (baseline − follow-up) for perceived cigarette prevalence in School 3 (N = 186).

(E-)cigarette Use	Contrast	Estimate	95% CI	SE	df	t ratio	p value
Never	T0 – T1	-0.455	−1.10 – 0.19	0.326	89.7	-1.40	0.167
Former	T0 – T1	-0.464	−2.04 – 1.11	0.797	141.1	-0.58	0.561
Occasional	T0 – T1	-0.597	−1.70 – 0.51	0.558	121.2	-1.07	0.287
Regular	T0 – T1	1.142	0.16 – 2.13	0.498	105.5	2.29	0.024

Note. Estimates represent differences between baseline (T0) and follow-up (T1). Confidence intervals are 95% and based on the Kenward–Roger degrees-of-freedom method. Never-smokers served as the reference category.

4.1.4 Sensitivity analysis

Table S17. Sensitivity analysis for School 3 perceived cigarette prevalence (adjusted for age and gender)

Section	Comparison / Predictor	Estimate / Test	95% CI	p
**Model comparison (ML)**	Timepoint × (E-)cigarette Use	$\chi^2(3) = 8.27$	–	0.041
**Final adjusted model (REML)**	Timepoint (post vs pre)*	0.11	−0.34 to 0.56	0.64

Note. Linear mixed-effects models with a random intercept for participant identification; adjusted for age (centered) and gender. Model comparison was conducted using maximum likelihood. The timepoint × (e-)cigarette use interaction remained statistically significant after adjustment, indicating that changes in perceived cigarette prevalence over time differed by (e-)cigarette use. Final estimates were obtained using restricted maximum likelihood. Never-smokers served as the reference category.

Perceived E-Cigarette Prevalence School 3

4.1.5 Model Comparison

Table S18. Likelihood-ratio test comparing full and reduced models predicting perceived e-cigarette prevalence in School 3 at baseline and follow-up (N = 186).

Model	df	AIC	BIC	logLik
Reduced Model	7	801.65	824.23	-393.82
Full Model	10	806.19	838.45	-393.10

Note. AIC = Akaike information criterion; BIC = Bayesian information criterion.

4.1.6 Model Output

Table S19. Reduced linear mixed-effects model predicting perceived e-cigarette prevalence in School 3 at baseline and follow-up (N = 186).

	**Perceived Prevalence (E-Cigarette)**			
Predictors	*Estimates*	*CI*	*Statistic*	*p*
(Intercept)	6.41	5.91 – 6.91	25.42	**<0.001**
Timepoint [T1]	0.29	-0.23 – 0.82	1.10	0.272
(E-)cigarette Use [Former]	0.68	-0.43 – 1.79	1.21	0.228
(E-)cigarette Use [Occasional]	0.53	-0.32 – 1.38	1.23	0.219
(E-)cigarette Use [Regular]	-0.11	-0.92 – 0.69	-0.27	0.784
**Random Effects**				
σ^2	1.78			
τ_{00} Identification	2.63			
ICC	0.60			
N Identification	161			
Observations	186			
Marginal R^2 / Conditional R^2	0.024 / 0.606			

Note. CI = confidence interval; ICC = intraclass correlation coefficient; σ^2 = residual variance; τ_{00} = variance of the random intercept. Never-smokers served as the reference category.

4.1.7 Sensitivity analysis

Table S20. Sensitivity analysis for School 3 perceived e-cigarette prevalence (adjusted for age and gender)

Section	Comparison / Predictor	Estimate / Test	95% CI	p
**Model comparison (ML)**	Timepoint × (E-)cigarette Use	$\chi^2(3) = 1.77$	–	0.62
**Final adjusted model (REML)**	Timepoint (post vs pre)	0.28	−0.22 to 0.79	0.28

Note. Linear mixed-effects models with a random intercept for participant identification; adjusted for age (centered) and gender. Model comparison was conducted using maximum likelihood. The timepoint × (e-)cigarette use interaction was not supported, indicating that changes in perceived e-cigarette prevalence over time did not differ by (e-)cigarette use. The main effect of timepoint was not statistically significant after adjustment. Never-smokers served as the reference category.

© 2026 Morf A. et al.
